# Exploring Breaks in Sedentary Behavior of Older Adults Immediately After Receiving Personalized Haptic Feedback: Intervention Study

**DOI:** 10.2196/26387

**Published:** 2021-05-10

**Authors:** Sofie Compernolle, Delfien Van Dyck, Greet Cardon, Ruben Brondeel

**Affiliations:** 1 Department of Movement and Sports Sciences Ghent University Ghent Belgium; 2 Research Foundation Flanders Brussels Belgium

**Keywords:** tactile feedback, sitting behavior, sedentary behavior, older adults, mHealth intervention, self-monitoring

## Abstract

**Background:**

“Push” components of mobile health interventions may be promising to create conscious awareness of habitual sedentary behavior; however, the effect of these components on the near-time, proximal outcome, being breaks in sedentary behavior immediately after receiving a push notification, is still unknown, especially in older adults.

**Objective:**

The aims of this study are to examine if older adults break their sedentary behavior immediately after receiving personalized haptic feedback on prolonged sedentary behavior and if the percentage of breaks differs depending on the time of the day when the feedback is provided.

**Methods:**

A total of 26 Flemish older adults (mean age 64.4 years, SD 3.8) wore a triaxial accelerometer (Activator, PAL Technologies Ltd) for 3 weeks. The accelerometer generated personalized haptic feedback by means of vibrations each time a participant sat for 30 uninterrupted minutes. Accelerometer data on sedentary behavior were used to estimate the proximal outcome, which was sedentary behavior breaks immediately (within 1, 3, and 5 minutes) after receiving personalized haptic feedback. Generalized estimating equations were used to investigate whether or not participants broke up their sedentary behavior immediately after receiving haptic feedback. A time-related variable was added to the model to investigate if the sedentary behavior breaks differed depending on the time of day.

**Results:**

A total of 2628 vibrations were provided to the participants during the 3-week intervention period. Of these 2628 vibrations, 379 (14.4%), 570 (21.7%), and 798 (30.4%) resulted in a sedentary behavior break within 1, 3 and 5 minutes, respectively. Although the 1-minute interval did not reveal significant differences in the percentage of breaks depending on the time at which the haptic feedback was provided, the 3- and 5-minute intervals did show significant differences in the percentage of breaks depending on the time at which the haptic feedback was provided. Concretely, the percentage of sedentary behavior breaks was significantly higher if personalized haptic feedback was provided between noon and 3 PM compared to if the feedback was provided between 6 and 9 AM (odds ratio 1.58, 95% CI 1.01-2.47, within 3 minutes; odds ratio 1.78, 95% CI 1.11-2.84, within 5 minutes).

**Conclusions:**

The majority of haptic vibrations, especially those in the morning, did not result in a break in the sedentary behavior of older adults. As such, simply bringing habitual sedentary behavior into conscious awareness seems to be insufficient to target sedentary behavior. More research is needed to optimize push components in interventions aimed at the reduction of the sedentary behavior of older adults.

**Trial Registration:**

ClinicalTrials.gov NCT04003324; https://clinicaltrials.gov/ct2/show/NCT04003324

## Introduction

Evidence shows that older adults (aged ≥60 years) are the most sedentary segment of the population [1]. They spend approximately 80% of their awake time (8-12 hours per day) in sedentary activities [2]. This finding is alarming, as prolonged sedentary behavior has been associated with increased risk for negative health outcomes, such as frailty, type 2 diabetes, and all-cause mortality [3]. To date, the number of interventions targeting sedentary behavior in older adults has been limited. Only 9 interventions could be detected in a systematic review [4], of which only 1 was a mobile health (mHealth) intervention [5]. This finding contrasts sharply with the range of mHealth interventions aimed at the promotion of physical activity [6], and it is also very disappointing, as a recent meta-analysis showed a significantly higher decrease in sedentary behavior following mHealth interventions compared to traditional interventions in all age groups [7]. The superiority of mHealth interventions over traditional interventions may be explained by the fact that a large amount of sedentary behavior is contextually triggered and automatic (ie, it involves little reasoning and is performed without conscious decision-making) [8]. In contrast, physical activity is regulated by controlled processes, such as intentions, values, and beliefs. Undesired automatic behavior can be disrupted by bringing the behavior and its context into conscious awareness—for example, by means of self-monitoring [9]. Self-monitoring can be easily integrated in mHealth interventions; therefore, these interventions offer great potential to reduce sedentary behavior.

mHealth interventions generally consist of multiple intervention components. Some are “pull” components, which require individuals to access the component on their mobile device at moments when they decide they need help. Others are “push” components, which are initiated by the intervention and are delivered via haptic vibrations, notifications, or text messages [10]. Previous efficacy studies mainly investigated whether the combination of pull and push components resulted in a reduction of total sedentary time at the end of the intervention. This reduction can be considered to be the desired distal outcome of the intervention. However, as push components may best facilitate the process of bringing habitual sedentary behavior and its context into conscious awareness [11,12], it may be more pertinent to investigate the near-time, proximal effect of these push components (ie, breaks in sedentary behavior immediately after receiving a push notification) compared to their effect on total sedentary time*.* To date, little effort has focused on examining these near-time, proximal effects [13]; only one study could be found in the literature [14]. In this study, 86 office workers received persuasive text messages to break up their sedentary behavior after 30 minutes of uninterrupted computer time. The results showed a steep decline in sedentary behavior in the 30 minutes following a text message compared to a control group [14]. To our knowledge, no studies are available on the proximal outcomes of push components aimed at the reduction of sedentary behavior in older adults.

Therefore, the aims of this exploratory study were (1) to examine if older adults break their sedentary behavior immediately after receiving personalized haptic feedback, and (2) to investigate if those breaks differed depending on the time of the day when the feedback was delivered. This investigation may be important, as push components that are offered at an inappropriate time may lead to burden and disengagement [15].

## Methods

### Study Design

This study reports on part of a larger mixed methods study being conducted to evaluate a self-monitoring mHealth intervention to reduce sedentary behavior in older adults [16]. The study was registered at ClinicalTrials.gov (identification number: NCT04003324) and was approved by the Committee of Medical Ethics of the Ghent University Hospital (Belgian registration number 2019/0398). All participants provided written informed consent.

### Participants, Procedure, and Intervention

Participant recruitment was conducted in Flanders between February and March 2019 using convenience sampling (ie, Facebook advertisements and an existing database [17]). To be eligible for the current study, participants needed to (1) be at least 60 years old, (2) be Dutch-speaking, (3) be able to walk 100 meters without severe difficulties, and (4) have a smartphone. A detailed description of the study procedure has been published elsewhere [16]. Briefly, baseline data, including sociodemographic characteristics, were collected before the start of the intervention. Subsequently, the self-monitoring mHealth intervention was introduced to the participants. The intervention consisted of general sedentary behavior information and visual and haptic feedback on the participants’ sedentary behavior. General sedentary behavior information was provided to participants by means of a 10-minute presentation. The presentation was given by an expert in the field during the second home visit. Visual and haptic feedback were provided using a novel, validated triaxial accelerometer—the Activator (PAL Technologies Ltd) [18]. The Activator was worn during waking hours on the front of the thigh, either in a pants pocket or attached with an elastic band to clothing covering the upper thigh (eg, trousers, jeans, shorts, leggings, tights, or dresses) [19]. Real-time visual feedback and a 7-day historical overview were presented through a smartphone app via a Bluetooth connection. Haptic feedback was provided by a strong but comfortable vibration of the Activator accelerometer itself each time a participant sat for 30 uninterrupted minutes. Participants were instructed to break up their sedentary behavior each time they received a haptic vibration.

### Measures

The participants’ sociodemographic characteristics were administered using a structured interview and included age, gender, family situation, educational level, weight, and height. Sedentary behavior after receiving personalized haptic feedback was collected using the Activator device. The Activator device collected triaxial accelerometer data about thigh position and accelerations and processed the data via proprietary algorithms (Intelligent Activity Classification, PAL Technologies) to determine the wearer’s body posture (ie, sitting/lying and upright) and stepping speed. Accelerometer data were stored on the cloud server of PAL Technologies and used to estimate the proximal outcome, which was a break in sedentary behavior immediately (within 1, 3, and 5 minutes) after receiving personalized haptic feedback. Time-related characteristics of when the haptic feedback was provided were extracted from the system usage data of the Activator and categorized into the following six categories: 6-9 AM, 9 AM-noon, noon-3 PM, 3-6 PM, 6-9 PM, 9 PM-midnight.

### Data Cleaning and Statistical Analyses

Descriptive statistics of the sample and of the personalized haptic feedback were summarized as proportions, means, and standard deviations. Participants’ sedentary behavior within 1, 3, and 5 minutes after receiving haptic feedback was extracted from the accelerometer data and dichotomized as whether or not participants had broken up their sedentary behavior. If a participant broke up their sedentary behavior in the first minute after receiving a notification, this was taken into account in all three time frames. As each participant received haptic feedback multiple times, observations were nested for the participants. Generalized estimating equations, including a random intercept, were applied to investigate whether participants broke up their sedentary behavior immediately after receiving haptic feedback. The time-related variable was added to the model as a fixed effect to investigate if the sedentary behavior breaks differed depending on the time of day. Nonstandardized regression coefficients (β) and 95% confidence intervals were reported as effect estimates. If sedentary behavior breaks were observed, proportions were requested to determine the duration of the breaks. Analyses were performed in SPSS, version 25 (IBM Corporation).

## Results

### Descriptive Statistics of the Participants

The participant characteristics are presented in Table 1. Half of the participants were female, and the average age was 64.4 years (SD 3.8). The majority of the participants were highly educated and were married or lived with a partner. The participants’ mean BMI was 25.2 kg/m^2^ (SD 3.8).

**Table 1:** Participant characteristics (N=26).

Sociodemographic characteristic		Value
**Gender**		
	Men, n (%)	13 (50)
	Women, n (%)	13 (50)
**Age (years)**		
	Mean (SD), range	64.4 (3.8), 60.0-76.0
	Young older adults (<65), n (%)	14 (54)
	Older adults (≥65), n (%)	12 (46)
**Educational level, n (%)**		
	Secondary education	11 (42)
	College or university	15 (58)
**Family situation, n (%)**		
	No partner (ie, single, widowed)	4 (15)
	Partner but living separately	1 (4)
	Married or living with a partner	21 (81)
**BMI (kg/m^2^)**		
	Mean (SD), range	25.2 (3.8), 19.7-32.3
	Healthy weight (<25), n (%)	16 (62)
	Overweight (25-29.9), n (%)	6 (23)
	Obese (>30), n (%)	4 (15)

### Descriptive Statistics of the Personalized Haptic Feedback

**Figure 1:**
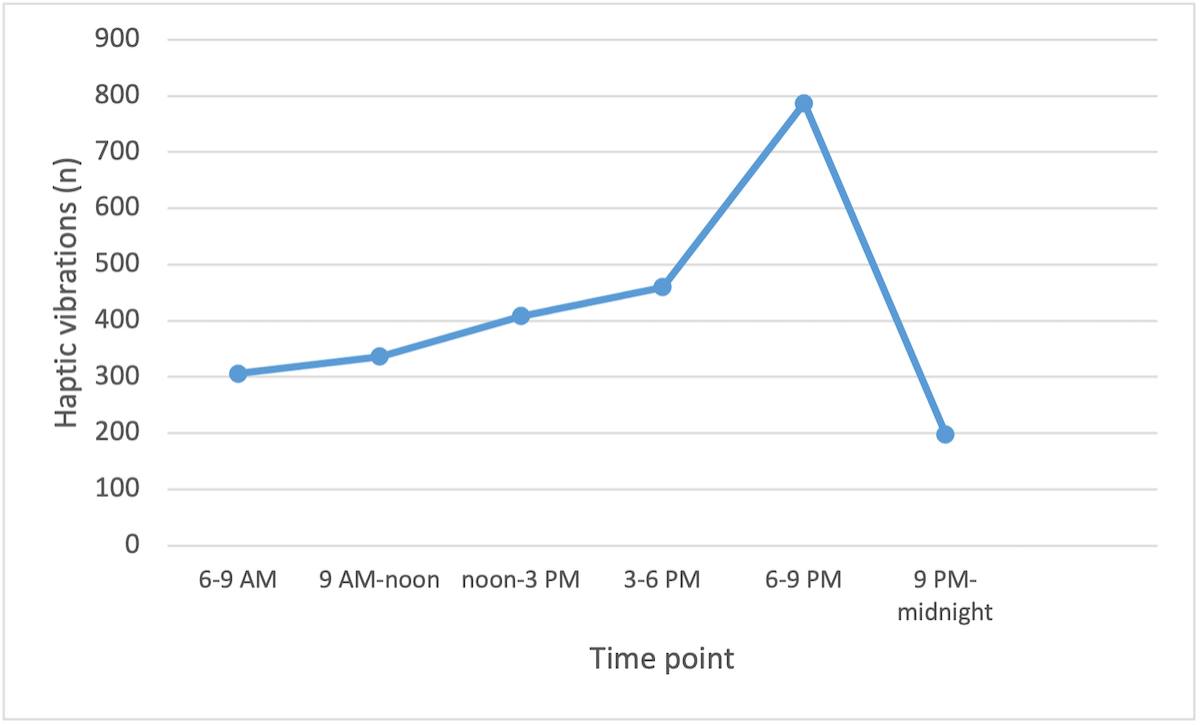
Number of haptic vibrations at different time points.

A total of 2628 vibrations were provided to the participants during the 3-week intervention period. The highest number of vibrations was provided between 6 and 9 PM, whereas the lowest number of vibrations was provided between 6 and 9 AM (306 and 787, respectively) (see Figure 1). Considerable differences were observed in the number of vibrations between participants, ranging from 3-258 vibrations during the 3-week intervention period. The median number of vibrations that participants received per day varied from 0-7. Detailed information on the participants’ personalized haptic feedback is provided in Multimedia Appendix 1.

### Breaks in the Sedentary Behavior of Older Adults Immediately After Receiving Personalized Haptic Feedback

**Figure 2:**
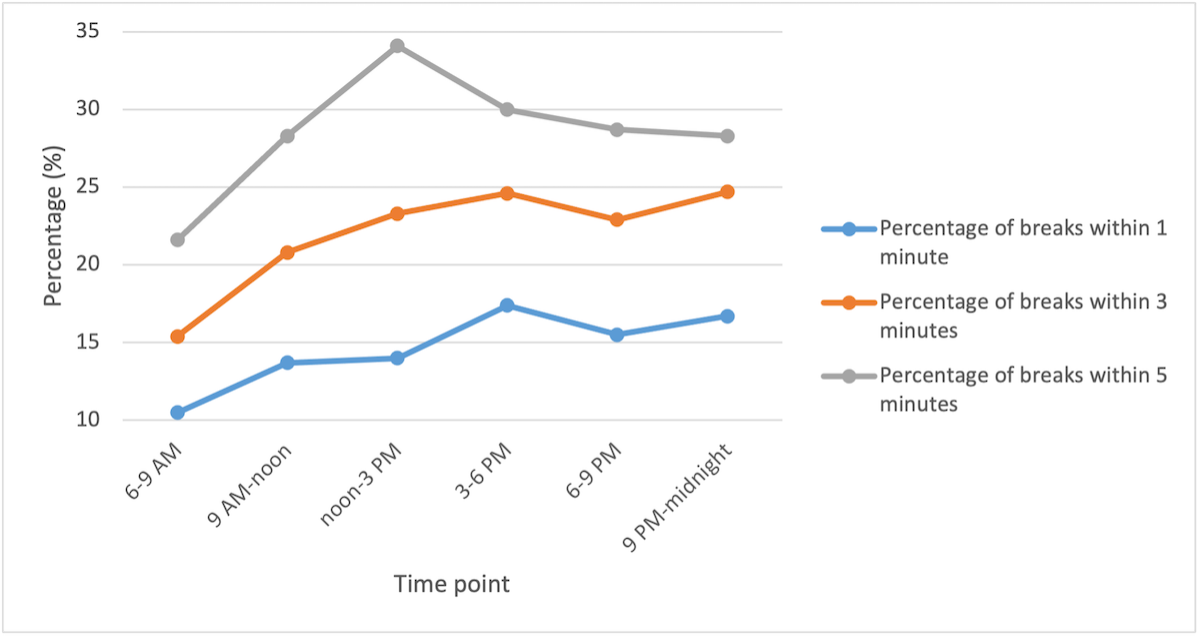
Percentages of vibrations followed by a break in sedentary behavior by time of day.

Of the 2628 personalized haptic vibrations, 379 (14.4%), 570 (21.7%), and 798 (30.4%) resulted in a sedentary behavior break within 1, 3, and 5 minutes, respectively. Although the 1-minute interval did not reveal significant differences in the percentage of breaks depending on the time at which the haptic feedback was provided, the 3 and 5-minute intervals did show significant differences in the percentage of breaks depending on the time at which the haptic feedback was provided. Concretely, the percentage of sedentary behavior breaks was significantly higher if personalized haptic feedback was provided between noon and 3 PM (3- and 5-minute intervals), compared to if the feedback was provided between 6 and 9 AM (see Table 2 and Figure 2). The duration of the breaks observed in the older adults’ sedentary behavior is summarized in Table 3.

**Table 2:** Sedentary behavior breaks by time of the day when the vibrations were provided.

Time of day	1 minute		3 minutes		5 minutes	
	OR^a^ (95% CI)	*P* value	OR (95% CI)	*P* value	OR (95% CI)	*P* value
6-9 AM	1.00 (reference)	N/A^b^	1.00 (reference)	N/A	1.00 (reference)	N/A
9 AM-noon	1.38 (0.75-2.54)	.31	1.46 (0.81-2.62)	.21	1.42 (0.80-2.54)	.23
Noon-3 PM	1.26 (0.78-2.03)	.34	*1.58 (1.01-2.47)* ^c^	*.05*	*1.78 (1.11-2.84)*	*.02*
3-6 PM	1.58 (0.85-2.94]	.15	1.62 (0.93-2.81)	.09	1.37 (0.80-2.36)	.25
6-9 PM	1.92 (0.74-2.25)	.36	1.41 (0.80-2.48)	.24	1.25 (0.71-2.21)	.44
9 PM-midnight	1.46 (0.62-3.44)	.39	1.66 (0.86-3.23)	.13	1.38 (0.67-2.87)	.38

^a^OR: odds ratio.

^b^N/A: not applicable.

^c^Italic text indicates the most significant time periods.

**Table 3:** Duration of the sedentary behavior breaks.

Duration of break (minutes)	Vibrations followed by a break within 3 minutes (n=546), n (%)	Vibrations followed by a break within 5 minutes (n=714), n (%)
<1	246 (45.1)	279 (38.7)
1-2	121 (22.1)	127 (17.6)
2-3	179 (32.8)	93 (12.9)
3-5	N/A^a^	215 (29.9)

^a^N/A: not applicable.

## Discussion

This is the first study investigating breaks in the sedentary behavior of older adults immediately after receiving personalized haptic feedback. The results are rather disappointing, as less than 1 in 3 vibrations resulted in a sedentary behavior break within 5 minutes. Moreover, more than half of the breaks lasted less than 2 minutes, which may be too short to achieve health benefits [20]. Consequently, it can be concluded that simply increasing awareness of habitual sedentary behavior by means of personalized haptic feedback is insufficient to stimulate older adults to break their sedentary behavior. Although underlying reasons to ignore the personalized haptic feedback were not examined, previous research showed that many older adults lack the motivation (or capabilities) to break their sedentary behavior [21]. Therefore, more effort should be made to enhance older adults’ motivation to break their sedentary behavior.

Our results also suggested that the percentage of breaks differed depending on the time of the day when the haptic feedback was provided (at least when analyzing the 3- and 5-minute intervals). Based on previous ecological momentary assessment studies, showing that older adults’ fatigue increases throughout the day [22,23], it was expected that participants would be more likely to respond to the haptic feedback in the morning compared to in the afternoon and the evening. However, our results showed the opposite. Older adults were more likely to break their sedentary behavior in the afternoon compared to in the morning. It is possible that the moments when the participants are sedentary in the morning are “necessary moments of rest,” as our results showed that they are much more active in the morning. On the other hand, in the afternoon, older adults are much more sedentary and probably thus more motivated to break their sedentary behavior after receiving a haptic vibration [1]. If this result can be replicated in future research, haptic vibrations, notifications, or text messages aimed at the reduction of older adults’ sedentary behavior should preferably be provided in the afternoon to be successful.

The main strength of the current study is its innovativeness. As far as we know, no previous studies have investigated older adults’ breaks in sedentary behavior immediately after receiving haptic feedback. The most relevant limitations are the small sample size and the simplicity of the study design. The small sample size hinders the investigation of individual differences (eg, sociodemographic and behavioral characteristics) between participants who often broke their sedentary behavior and those who did not. The simplicity of the study design prevents us from drawing firm conclusions on the causality of the association between the push components and breaks in sedentary behavior. As a vibration was provided each time a participant was sedentary for 30 minutes, it remains unclear what the response would have been (ie, break or no break) if no vibration was given. Therefore, the use of micro-randomized trials is recommended to confirm and further elaborate the current findings.

### Conclusion

The majority of haptic vibrations, especially those received in the morning, did not result in a break in older adults’ sedentary behavior. As such, simply bringing habitual sedentary behavior into conscious awareness seems to be insufficient to target sedentary behavior. More research is needed to optimize push components in interventions aimed at the reduction of sedentary behavior in older adults.

## Appendix

Multimedia Appendix 1 Number of vibrations per participant per day.

## Abbreviations

mHealth: mobile health

## References

[1] Leask CF, Harvey JA, Skelton DA, Chastin SF (2015). Exploring the context of sedentary behaviour in older adults (what, where, why, when and with whom). Eur Rev Aging Phys Act.

[2] Matthews CE, Chen KY, Freedson PS, Buchowski MS, Beech BM, Pate RR, Troiano RP (2008). Amount of time spent in sedentary behaviors in the United States, 2003-2004. Am J Epidemiol.

[3] de Rezende Leandro Fornias Machado, Rodrigues Lopes M, Rey-López Juan Pablo, Matsudo VKR, Luiz ODC (2014). Sedentary behavior and health outcomes: an overview of systematic reviews. PLoS One.

[4] Chastin S, Gardiner P, Ashe M, Harvey J, Leask C, Balogun S, Helbostad J, Skelton D (2017). Interventions for reducing sedentary behaviour in community-dwelling older adults. Cochrane Database Syst Rev.

[5] Lyons EJ, Swartz MC, Lewis ZH, Martinez E, Jennings K (2017). Feasibility and acceptability of a wearable technology physical activity intervention with telephone counseling for mid-aged and older adults: a randomized controlled pilot trial. JMIR mHealth uHealth.

[6] Elavsky S, Knapova L, Klocek A, Smahel D (2019). Mobile health interventions for physical activity, sedentary behavior, and sleep in adults aged 50 years and older: a systematic literature review. J Aging Phys Act.

[7] Direito A, Carraça Eliana, Rawstorn J, Whittaker R, Maddison R (2017). mHealth technologies to influence physical activity and sedentary behaviors: behavior change techniques, systematic review and meta-analysis of randomized controlled trials. Ann Behav Med.

[8] Maher JP, Conroy DE (2016). A dual-process model of older adults' sedentary behavior. Health Psychol.

[9] Hermsen S, Frost J, Renes RJ, Kerkhof P (2016). Using feedback through digital technology to disrupt and change habitual behavior: a critical review of current literature. Computers in Human Behavior.

[10] Luers B, Klasnja P, Murphy S (2019). Standardized effect sizes for preventive mobile health interventions in micro-randomized trials. Prev Sci.

[11] Compernolle S, DeSmet A, Poppe L, Crombez G, De Bourdeaudhuij I, Cardon G, van der Ploeg HP, Van Dyck D (2019). Effectiveness of interventions using self-monitoring to reduce sedentary behavior in adults: a systematic review and meta-analysis. Int J Behav Nutr Phys Act.

[12] Conroy DE, Maher JP, Elavsky S, Hyde AL, Doerksen SE (2013). Sedentary behavior as a daily process regulated by habits and intentions. Health Psychol.

[13] Klasnja P, Hekler EB, Shiffman S, Boruvka A, Almirall D, Tewari A, Murphy SA (2015). Microrandomized trials: an experimental design for developing just-in-time adaptive interventions. Health Psychol.

[14] van Dantzig S, Geleijnse G, van Halteren AT (2012). Toward a persuasive mobile application to reduce sedentary behavior. Pers Ubiquit Comput.

[15] Walton A, Nahum-Shani I, Crosby L, Klasnja P, Murphy S (2018). Optimizing digital integrated care via micro-randomized trials. Clin Pharmacol Ther.

[16] Compernolle S, Cardon G, van der Ploeg HP, Van Nassau F, De Bourdeaudhuij I, Jelsma JJ, Brondeel R, Van Dyck D (2020). Engagement, acceptability, usability, and preliminary efficacy of a self-monitoring mobile health intervention to reduce sedentary behavior in Belgian older adults: mixed methods study. JMIR mHealth uHealth.

[17] Lakerveld J, Ben Rebah M, Mackenbach JD, Charreire H, Compernolle S, Glonti K, Bardos H, Rutter H, De Bourdeaudhuij I, Brug J, Oppert J (2015). Obesity-related behaviours and BMI in five urban regions across Europe: sampling design and results from the SPOTLIGHT cross-sectional survey. BMJ Open.

[18] Gill JMR, Hawari NSA, Maxwell DJ, Louden D, Mourselas N, Bunn C, Gray CM, van der Ploeg HP, Hunt K, Martin A, Wyke S, Mutrie N (2018). Validation of a Novel Device to Measure and Provide Feedback on Sedentary Behavior. Med Sci Sports Exerc.

[19] Jelsma JGM, Renaud LR, Huysmans MA, Coffeng JK, Loyen A, van Nassau F, Bosmans JE, Speklé EM, van der Beek AJ, van der Ploeg HP (2019). The Dynamic Work study: study protocol of a cluster randomized controlled trial of an occupational health intervention aimed at reducing sitting time in office workers. BMC Public Health.

[20] Kehler DS, Clara I, Hiebert B, Stammers AN, Hay J, Schultz A, Arora RC, Tangri N, Duhamel TA (2019). The association between patterns of physical activity and sedentary time with frailty in relation to cardiovascular disease. Aging Med (Milton).

[21] Compernolle S, De Cocker K, Cardon G, De Bourdeaudhuij I, Van Dyck D (2020). Older adults' perceptions of sedentary behavior: a systematic review and thematic synthesis of qualitative studies. Gerontologist.

[22] Dunton GF, Atienza AA, Castro CM, King AC (2009). Using ecological momentary assessment to examine antecedents and correlates of physical activity bouts in adults age 50+ years: a pilot study. Ann Behav Med.

[23] Curran SL, Beacham AO, Andrykowski MA (2004). Ecological momentary assessment of fatigue following breast cancer treatment. J Behav Med.

