# Supplementary material for: Exploring Breaks in Sedentary Behavior of Older Adults Immediately After Receiving Personalized Haptic Feedback: Intervention Study
Source: JMIR Mhealth Uhealth. 2021 May 10;9(5):e26387. doi: 10.2196/26387 (PMC8145090; doi:10.2196/26387)
Supplement: Multimedia Appendix 1 [file mhealth_v9i5e26387_app1.docx]

Supplementary file 1: Number of vibrations per participant per day

|  | Day1 | Day2 | Day3 | Day4 | Day5 | Day6 | Day7 | Day8 | Day9 | Day10 | Day11 | Day12 | Day13 | Day14 | Day15 | Day16 | Day17 | Day18 | Day19 | Day20 | Day21 |
| --- | --- | --- | --- | --- | --- | --- | --- | --- | --- | --- | --- | --- | --- | --- | --- | --- | --- | --- | --- | --- | --- |
| 1 | 4 | 1 | 1 | 4 | 3 | 3 | 2 | 2 | 3 | 1 | 4 | 2 | 5 | 5 | 2 | 3 | 3 | 1 | 2 | 0 | 2 |
| 2 | 0 | 6 | 3 | 7 | 2 | 1 | 5 | 2 | 4 | 10 | 3 | 10 | 10 | 0 | 3 | 7 | 5 | 8 | 8 | 8 | 10 |
| 3 | 1 | 8 | 9 | 6 | 8 | 0 | 6 | 4 | 9 | 1 | 10 | 10 | 10 | 6 | 3 | 7 | 1 | 5 | 8 | 5 | 2 |
| 4 | 0 | 0 | 0 | 4 | 0 | 5 | 10 | 2 | 3 | 1 | 4 | 5 | 4 | 4 | 3 | 0 | 1 | 3 | 4 | 5 | 1 |
| 5 | 2 | 1 | 7 | 4 | 7 | 4 | 4 | 7 | 6 | 10 | 4 | 10 | 4 | 10 | 8 | 9 | 12 | 10 | 8 | 9 | 7 |
| 6 | 3 | 3 | 3 | 4 | 3 | 0 | 3 | 3 | 3 | 5 | 4 | 2 | 2 | 0 | 0 | 2 | 1 | 0 | 2 | 0 | 1 |
| 7 | 5 | 8 | 10 | 4 | 0 | 1 | 0 | 3 | 0 | 3 | 11 | 0 | 9 | 7 | 11 | 13 | 14 | 8 | 2 | 2 | 10 |
| 8 | 0 | 2 | 6 | 4 | 7 | 6 | 3 | 5 | 5 | 1 | 1 | 0 | 6 | 6 | 1 | 3 | 4 | 3 | 1 | 2 | 5 |
| 9 | 2 | 0 | 0 | 3 | 3 | 2 | 7 | 7 | 1 | 3 | 2 | 1 | 4 | 6 | 3 | 4 | 0 | 2 | 1 | 2 | 2 |
| 10 | 1 | 2 | 3 | 3 | 6 | 2 | 5 | 3 | 8 | 6 | 6 | 8 | 4 | 4 | 8 | 0 | 9 | 7 | 3 | 2 | 7 |
| 11 | 3 | 3 | 2 | 4 | 3 | 8 | 3 | 0 | 5 | 9 | 2 | 7 | 5 | 6 | 5 | 4 | 6 | 4 | 10 | 5 | 8 |
| 12 | 1 | 1 | 2 | 4 | 7 | 3 | 2 | 3 | 2 | 1 | 1 | 1 | 0 | 2 | 0 | 2 | 1 | 2 | 2 | 1 | 0 |
| 13 | 3 | 7 | 2 | 4 | 6 | 6 | 5 | 5 | 0 | 3 | 6 | 3 | 5 | 3 | 5 | 2 | 0 | 1 | 0 | 0 | 5 |
| 14 | 3 | 8 | 3 | 3 | 2 | 1 | 8 | 6 | 2 | 5 | 0 | 3 | 10 | 5 | 3 | 12 | 12 | 1 | 7 | 4 | 5 |
| 15 | 3 | 0 | 3 | 0 | 1 | 3 | 2 | 3 | 3 | 0 | 2 | 6 | 2 | 3 | 1 | 6 | 3 | 1 | 0 | 0 | 4 |
| 16 | 2 | 2 | 2 | 1 | 2 | 1 | 1 | 3 | 0 | 6 | 3 | 1 | 2 | 3 | 0 | 4 | 6 | 2 | 1 | 2 | 5 |
| 17 | 1 | 0 | 2 | 0 | 2 | 1 | 0 | 0 | 0 | 2 | 2 | 0 | 0 | 1 | 2 | 1 | 0 | 2 | 4 | 1 | 2 |
| 18 | 0 | 2 | 0 | 4 | 4 | 1 | 1 | 3 | 6 | 4 | 4 | 2 | 5 | 2 | 2 | 3 | 1 | 5 | 3 | 0 | 4 |
| 19 | 4 | 2 | 2 | 0 | 1 | 3 | 0 | 11 | 3 | 4 | 8 | 10 | 2 | 0 | 0 | 0 | 1 | 1 | 5 | 0 | 6 |
| 20 | 3 | 1 | 2 | 1 | 2 | 3 | 2 | 2 | 1 | 4 | 3 | 1 | 1 | 0 | 4 | 5 | 4 | 3 | 5 | 4 | 0 |
| 21 | 6 | 1 | 0 | 4 | 2 | 0 | 0 | 0 | 1 | 0 | 0 | 0 | 1 | 1 | 2 | 2 | 2 | 2 | 0 | 2 | 0 |
| 22 | 2 | 1 | 3 | 2 | 2 | 1 | 5 | 6 | 0 | 4 | 3 | 1 | 2 | 3 | 1 | 1 | 1 | 10 | 6 | 5 | 1 |
| 23 | 1 | 1 | 1 | 2 | 2 | 1 | 0 | 0 | 1 | 1 | 4 | 2 | 1 | 0 | 0 | 1 | 2 | 3 | 4 | 0 | 0 |
| 24 | 4 | 7 | 1 | 1 | 3 | 6 | 5 | 3 | 8 | 14 | 13 | 0 | 7 | 5 | 2 | 1 | 0 | 2 | 0 | 1 | 4 |
| 25 | 0 | 0 | 1 | 2 | 0 | 1 | 0 | 0 | 0 | 0 | 0 | 0 | 1 | 0 | 0 | 0 | 0 | 0 | 0 | 1 | 0 |
| 26 | 2 | 2 | 2 | 4 | 2 | 2 | 3 | 3 | 3 | 3 | 3 | 2 | 4 | 3 | 2 | 3 | 2 | 2 | 3 | 2 | 4 |
